# Translation, validation and cultural adaptation of “The Eustachian Tube Dysfunction Questionnaire-7” (ETDQ-7) to Brazilian Portuguese (BR)^☆^

**DOI:** 10.1016/j.bjorl.2018.03.010

**Published:** 2018-04-19

**Authors:** Fernanda Pires Gallardo, Ektor Tsuneo Onishi, Francisco Iure Lira, Flávia Barros Suzuki, José Ricardo Gurgel Testa

**Affiliations:** Universidade Federal de São Paulo (UNIFESP), São Paulo, SP, Brazil

**Keywords:** Eustachian tube/pathophysiology, Otitis media/diagnosis, Surveys and questionnaires, Translation, Eustachian tube/therapy, Tuba auditiva/fisiopatologia, Otite média/diagnóstico, Inquéritos e questionários, Tradução, Tuba auditiva/terapia

## Abstract

**Introduction:**

Chronic Eustachian tube dysfunction can cause several symptoms and middle ear conditions that can impact patient quality of life. It is estimated to be relatively frequent, affecting approximately 5% of adults. The diagnostic tools for this condition are still inadequate. In 2012, McCoul et al. published a questionnaire for the evaluation of Eustachian tube dysfunction named ETDQ-7. They established its replicability and validity. The cutoff point for the diagnosis of chronic Eustachian tube dysfunction was equal to or greater than 14.5, with 100% sensitivity and 100% specificity.

**Objective:**

To translate, adapt and validate the ETDQ-7 questionnaire to Brazilian Portuguese.

**Methods:**

We translated the questionnaire into Brazilian Portuguese and applied it to 50 patients, 20 of whom had chronic Eustachian tube dysfunction, and 30 controls.

**Results:**

The results obtained with the North-American questionnaire were confirmed in its Brazilian version. The cut-off point for the diagnosis of chronic Eustachian tube dysfunction was ≥14, also exhibiting high sensitivity and specificity, very similar to that of ETDQ-7.

**Conclusion:**

It is recommended that ETDQ-7 be used to complement the clinical history of patients with chronic Eustachian tube dysfunction; it can also be used as an important tool for diagnosis, patient follow-up and treatment management.

## Resumo

**Introdução:**

A disfunção crônica da tuba auditiva pode causar diversos sintomas e doenças de orelha média e impactar a qualidade de vida dos pacientes. Estima-se que é uma morbidade relativamente frequente, acomete cerca de 5% dos adultos. Os instrumentos diagnósticos para essa afecção ainda são insuficientes. Em 2012 McCoul et al. publicaram um questionário para a avaliação da disfunção da tuba auditiva denominado ETDQ-7. Eles demonstraram sua reprodutibilidade e validade. O ponto de corte para o diagnóstico de disfunção de crônica da tuba auditiva foi de maior ou igual a 14,5 com 100% de sensibilidade e 100% de especificidade.

**Objetivo:**

Traduzir, adaptar e validar o questionário ETDQ-7 para o português brasileiro.

**Método:**

Traduzimos o questionário para o português do Brasil e o aplicamos em 50 pacientes, 20 com disfunção crônica da tuba auditiva e 30 controles.

**Resultados:**

Os resultados obtidos no questionário americano foram confirmados na versão brasileira. O ponto de corte para o diagnóstico de disfunção crônica da tuba auditiva foi maior ou igual a 14, também com alta sensibilidade e especificidade, muito semelhante ao ETDQ-7.

**Conclusão:**

Recomenda-se que o ETDQ-7 seja usado como um complemento na história clínica do paciente com disfunção crônica da tuba auditiva e pode ser usado também como uma importante ferramenta para fins de diagnóstico, seguimento de pacientes e manejos no tratamento.

## Introduction

The middle ear is an aerated cavity within the petrous portion of the temporal bone, that is periodically ventilated when the Eustachian Tube (ET) opens. The ET is situated between the nasopharynx and the Middle Ear (ME). Its main function is to equalize the middle ear pressure with that of the external environment when necessary through the opening of its ostium in the nasopharynx.^1^ Among other functions is the middle ear protection and the clearance of middle ear secretions to the nasopharynx.^1^, ^2^, ^3^

Eustachian tube dysfunction (ETD) exists when there is a failure in the tubal mechanism to open or close properly, leading to ME pressure deregulation, nasopharyngeal secretion reflux into the tympanic cavity and impairment of drainage of ME contents into the nasopharynx.^4^ This dysfunction can cause several otologic pathologic processes, leading to symptoms such as otalgia, aural fullness or sensation of pressure in the ears, hearing loss, tinnitus, vertigo, and imbalance, and others.^4^, ^5^, ^6^

ETD is common and affects up to 5% of adults. When chronic (symptom persistence for more than 3 months) it can have a significant impact on the quality of life.^1^ This condition has a wide variety of signs and symptoms.

The clinical evaluation of these symptoms is subjective, based essentially on patients’ complaints, physical examination and some ancillary tests, such as tympanometry or pneumatic otoscopy, that help to establish the diagnosis, but there is no tool to provide objective measurement of symptom intensity and morbidity,^7^ or to allow comparisons before and after clinical or surgical treatments.

Considering chronic ETD as a disease with an impact on the affected individual's quality of life, and taking into account that the diagnosis is mainly clinical and that several new treatments for this condition have been proposed, McCoul et al., in 2012, developed a questionnaire to assess the symptoms of chronic ETD, consisting of seven items with a scale of graduated responses ranging from 1 to 7, with “1” corresponding to the absence of the suggested symptom and “7”, to maximum symptom severity. This questionnaire is known as “Eustachian Tube Dysfunction Questionnaire-7” (ETDQ-7). It was developed based mainly on other questionnaires known in the literature, such as the OM-6 (Otitis Media-6 Item Quality-of-life), SNOT-20 (20-Item Sino-Nasal Outcomes Test), among others. Its validity, reproducibility and accountability have been previously demonstrated.^8^

Similar to quality of life questionnaires, symptom scores allow the quantitative measurement of subjective questions and have advantages over the clinical history alone. These scores can provide a more accurate estimate of the assessed disease morbidity, as well as information not identified by the physician during the usual anamnesis. Additionally, they allow for a formal and valid documentation of patient history for recording purposes and subsequent comparisons after the proposed treatments.^9^

As this questionnaire was originally written in English, to use it in our country we need to translate it into Brazilian Portuguese. However, a simple translation may not be effective, due to cultural differences between peoples. Therefore, it is necessary to perform the translation, cultural adaptation and validation of the ETDQ-7 for the Brazilian reality.

### Objective

To translate, adapt and validate the ETDQ-7 questionnaire into Brazilian Portuguese.

## Methods

### Ethical concerns

All ethical care was taken into account, based on Resolution 466/12 of the National Health Council. This project was approved by the Research Ethics Committee of Universidade Federal de São Paulo (UNIFESP) – Escola Paulista de Medicina (EPM) under number 1690/2016, and the Free and Informed Consent Form (FICF) was obtained, which was written utilizing easily understandable and objective language and signed by all participants involved in the study.

### Place

This study was developed at the Postgraduate Program in Otorhinolaryngology, UNIFESP/EPM, at the Otorhinolaryngology Outpatient Clinic of Hospital São Paulo, with the authorization of the Head of the Outpatient Clinic and the teaching and research coordinator of Hospital São Paulo.

### Sample

Fifty patients were evaluated in an observational, descriptive, analytical and cross-sectional study at the Otorhinolaryngology Outpatient Clinic of the Hospital (EPM/UNIFESP), from July 2016 to March 2017.

### Procedures

In the first phase of the study, the ETDQ-7 questionnaire was translated into Brazilian Portuguese. This translation required five main steps: (1) Translation; (2) Back-translation; (3) Review by a translation and back-translation committee; (4) Pre-test of equivalence by bilingual individuals and (5) Re-assessment of the weight of the scores, if relevant, as proposed by Guillemin.^10^ The translation and back-translation phases were performed by two bilingual individuals. The translation and back-translation committee consisted of the same individuals who carried out the abovementioned phases and a third one, who was also bilingual. After the final conciliation of the versions, the questions were culturally adapted so that they would become clearer and more understandable to patients. The questionnaire consists of 7 items, having a minimum score of 7 points and a maximum of 49 points (Table 1). After the translation was finished, the recruitment phase started.

**Table 1:** Questionnaire for evaluation of Eustachian Tube Dysfunction consisting of 7 items, translated into Brazilian Portuguese.

Original questionnaire							
During the past 1 month, how much of a problem was each of the following?	No problem		Moderate problem			Severe problem	
1. Pressure in the ears?	1	2	3	4	5	6	7
2. Pain in the ears?	1	2	3	4	5	6	7
3. A feeling that your ears are clogged or “under water”?	1	2	3	4	5	6	7
4. Ear problems when you have a cold or sinusitis?	1	2	3	4	5	6	7
5. Crackling or popping sounds in the ears?	1	2	3	4	5	6	7
6. Ringing in the ears?	1	2	3	4	5	6	7
7. A feeling that your hearing is muffled?	1	2	3	4	5	6	7

Original questionnaire.

Fifty patients were evaluated in an observational, descriptive, analytical and cross-sectional study carried out at the Otorhinolaryngology Outpatient Clinic of Hospital São Paulo (EPM/UNIFESP), from July 2016 to March 2017.

All patients were aged 18 years or older, agreed to participate in the study and signed the institutional Ethics and Research Committee's FICF, which was written using objective and easily understood language.

All patients underwent a complete anamnesis, including degree of schooling; considering that an adequate understanding of the questionnaire was essential, the translated ETDQ-7 questionnaire was used. All subjects also received a complete otorhinolaryngological clinical examination, fiberoptic nasopharyngolaryngoscopy and tympanometry. Thirty days after the first consultation, the same patients were recruited to fill out the questionnaire again and were submitted to a complete otorhinolaryngological examination and fiberoptic nasolaryngoscopy, without tympanometry at that time and without treatment during that period (similar to the original study design).

The inclusion criteria were:

*Chronic ETD group*: Patients with clinical symptoms of ETD for more than 3 months, having at least two of the following symptoms in one or both ears in the last month: sensation of aural fullness or pressure, hearing loss or muffled hearing, recurrent or persistent middle ear effusion or inability to adjust ear pressure after changes in atmospheric pressure. Otoscopy showing tympanic retraction and/or presence of retrotympanic fluid and tympanometry compatible with negative pressure in the middle ear (type B or C tympanometry curve tracings). The alterations in tympanometry were used as the gold standard for patient selection.
*Control group*: Patients without ETD complaints, with otorhinolaryngological examination and normal fiberoptic nasopharyngolaryngoscopy. The tympanometry without alterations (type A tympanometry curve tracings) was used as the gold standard to verify the absence of ETD in these patients.

Exclusion criteria were: patients younger than 18 years of age, patient refusal to participate in the study and/or refusal to sign the FICF, ETD symptoms for less than 3 months, presence of tympanic membrane lesions (such as granulomas, polyps and tympanosclerosis), which could affect the examination results, head and neck surgery in the previous three months, head and neck radiation therapy, history of tumors in the region, signs of acute sinonasal diseases, adenoid hypertrophy, craniofacial syndromes, including Down's Syndrome, palatine fissures, ciliary dyskinesias, or other systemic immunodeficiencies.

### Tools

In addition to the clinical otorhinolaryngological examination, all patients underwent fiberoptic nasopharyngolaryngoscopy (Scholly LUT, serial number: 354959) and tympanometry (Interacustic model Az 7), of which devices belonged to the Otorhinolaryngology Outpatient Clinic of the Hospital São Paulo.

### Statistical method

The statistical analysis of all the data collected in this study was initially carried out using descriptive statistics through mean, median, minimum and maximum values, standard deviation, absolute and relative frequencies (percentage), as well as the two-dimensional scatter plot.

The reliability study of the ETDQ-7 (Seven-item Eustachian Tube Dysfunction Questionnaire) regarding the test–retest aspect was carried out using Goodman and Kruskal gamma (*γ*) coefficients^11^ and Spearman's correlation.^11^ Internal consistency was assessed through Cronbach's alpha coefficient^12^ and the discriminatory validity between patient and control was assessed through the Receiver Operating Characteristic Curve (ROC) curve.

The level of significance *α* = 5% was used for all the conclusions obtained at the statistical analyses. The data were entered into Excel 2010 for Windows spreadsheets for the adequate storage of the information. Statistical analyses were carried out using the statistical program R version 3.3.2.

## Results

The sample selected for this study consisted of 50 individuals, 20 (40.0%) patients and 30 (60.0%) controls (Table 2).

**Table 2:** Distribution of the main characteristics in the patient and control groups.

	Patient		Control		Total	
*Gender*						
Male	8	40%	11	36.7%	19	38%
Female	12	60%	19	63.3%	31	62%
Total	20	100%	30	100%	50	100%

*Age (years)*						
*n*	20		30		50	
Mean	43.0		41.5		42.1	
Median	39.5		35.0		36.0	
Minimum	18		28		18	
Maximum	74		78		78	
Standard deviation	15.2		13.8		14.2	

*Level of schooling*						
Elementary School, incomplete	4	20%	3	10%	7	14%
Elementary School, complete	4	20%	4	13.3%	8	16%
High School, incomplete	–	–	1	3.3%	1	2.0%
High School, complete	7	35%	3	10%	10	20%
College or University, complete	5	25%	19	63.3%	24	48%
Total	20	100%	30	100%	50	100%

The patients’ group comprised 8 (40.0%) men and 12 (60.0%) women. Their mean age was 43 years, ranging from 18 to 74 years, with a standard deviation of 15.2 years. Most of them, 12 (60%) patients, had finished High School or College/University. Regarding the type of tympanometry curve tracing, type B was observed in 12 (60%) patients and type C, in 8 (40.0%) patients.

The control group consisted of 11 (36.7%) men and 19 (63.3%) women. Their mean age was 41.5 years, ranging from 28 to 78 years, with a standard deviation of 13.8 years. Most of them, 22 individuals (73.3%), had finished High School or College/University. All subjects in this group had bilateral type A tympanometry curve tracings.

The validation of the ETDQ-7 (The Seven-item Eustachian Tube Dysfunction Questionnaire) was an important research object of this study.

Table 3, Table 4, Table 5 disclose the answers of patients and controls to this questionnaire at two different times: in the beginning (“before”) and one month later (“after”). The reapplication of the questionnaire was intended to investigate the “test–retest reliability”. As summarized in Table 6, we observed a significant reliability of the questionnaire in the test–retest, since the estimates of the gamma (*γ*) coefficients of Goodman and Kruskal were close to 1 (one).

**Table 3:** Distribution of all individuals’ answers to the ETDQ-7 questionnaire, before and after.

	No problem				Moderate problem						Severe problem			
	1		2		3		4		5		6		7	
*Question 1*														
Before	25	50%	6	12%	4	8.0%	3	6.0%	3	6.0%	5	10%	4	8.0%
After	26	52%	6	12%	3	6.0%	2	4.0%	3	6.0%	8	16%	2	4.0%

*Question 2*														
Before	37	74%	6	12%	2	4.0%	3	6.0%	1	2.0%	–	–	1	2.0%
After	39	78%	6	12%	3	6.0%	1	2.0%	–	–	1	2.0%	–	–

*Question 3*														
Before	34	68%	2	4.0%	1	2.0%	4	8.0%	3	6.0%	2	4.0%	4	8.0%
After	33	66%	3	6.0%	4	8.0%	4	8.0%	1	2.0%	4	8.0%	1	2.0%

*Question 4*														
Before	24	48%	6	12%	4	8.0%	5	10%	1	2.0%	6	12%	4	8.0%
After	23	46%	6	12%	4	8.0%	4	8.0%	3	6.0%	5	10%	5	10%

*Question 5*														
Before	36	72%	6	12%	1	2.0%	–	–	1	2.0%	3	6.0%	3	6.0%
After	35	70%	6	12%	2	4.0%	–	–	3	6.0%	1	2.0%	3	6.0%

*Question 6*														
Before	25	50%	8	16%	6	12	–	–	2	4.0%	1	2.0%	8	16%
After	28	56%	7	14%	4	8.0%	1	2.0%	2	4.0%	4	8.0%	4	8.0%

*Question 7*														
Before	29	58%	5	10%	3	6.0%	5	10%	4	8.0%	2	4.0%	2	4.0%
After	29	58%	5	10%	4	8.0%	3	6.0%	3	6.0%	4	8.0%	2	4.0%

Question 1, Pressure in the ears; Question 2, Pain in the ears; Question 3, A feeling that your ears are clogged or “under water”; Question 4, Ear problems when you have a cold or sinusitis; Question 5, Crackling or popping sounds in the ears; Question 6, Ringing in the ears; Question 7, A feeling that your hearing is muffled.

**Table 4:** Distribution of patients’ answers to the ETDQ-7 questionnaire, before and after.

	No problem				Moderate problem						Severe problem			
	1		2		3		4		5		6		7	
*Question 1*														
Before	2	10%	2	10%	2	10%	2	10%	3	15%	5	25%	4	20%
After	–	–	4	20%	1	5.0%	2	10%	3	15%	8	40%	2	10%

*Question 2*														
Before	9	45%	5	25%	1	5.0%	3	15%	1	5.0%	–	–	1	5.0%
After	11	55%	5	25%	2	10%	1	5.0%	–	–	1	5.0%	–	–

*Question 3*														
Before	5	25%	1	5.0%	1	5.0%	4	20%	3	15%	2	10%	4	20%
After	3	15%	3	15%	4	20%	4	20%	1	5.0%	4	20%	1	5.0%

*Question 4*														
Before	2	10%	2	10%	1	5.0%	5	25%	1	5.0%	6	30%	3	15%
After	–	–	2	10%	2	10%	4	20%	3	15%	4	20%	5	25%

*Question 5*														
Before	9	45%	4	20%	–	–	–	–	1	5.0%	3	15%	3	15%
After	8	40%	4	20%	1	5.0%	–	–	3	15%	1	5.0%	3	15%

*Question 6*														
Before	3	15%	4	20%	2	10%	–	–	2	10%	1	5.0%	8	40%
After	5	25%	3	15%	1	5.0%	1	5.0%	2	10%	4	20%	4	20%

*Question 7*														
Before	4	20%	2	10%	2	10%	4	20%	4	20%	2	10%	2	10%
After	4	20%	1	5.0%	4	20%	2	10%	3	15%	4	20%	2	10%

Question 1, Pressure in the ears; Question 2, Pain in the ears; Question 3, A feeling that your ears are clogged or “under water”; Question 4, Ear problems when you have a cold or sinusitis; Question 5, Crackling or popping sounds in the ears; Question 6, Ringing in the ears; Question 7, A feeling that your hearing is muffled.

**Table 5:** Distribution of controls’ answers to the ETDQ-7 questionnaire, before and after.

	No problem				Moderate problem						Severe problem			
	1		2		3		4		5		6		7	
*Question 1*														
Before	23	76.7%	4	13.3%	2	6.7%	1	3.3%	–	–	–	–	–	–
After	26	86.7%	2	6.7%	2	6.7%	–	–	–	–	–	–	–	–

*Question 2*														
Before	28	93.3%	1	3.3%	1	3.3%	–	–	–	–	–	–	–	–
After	28	93.3%	1	3.3%	1	3.3%	–	–	–	–	–	–	–	–

*Question 3*														
Before	29	96.7%	1	3.3%	–	–	–	–	–	–	–	–	–	–
After	30	100%	–	–	–	–	–	–	–	–	–	–	–	–

*Question 4*														
Before	22	73.3%	4	13.3%	3	10.0%	–	–	–	–	–	–	1	3.3%
After	23	76.7%	4	13.3%	2	6.7%	–	–	–	–	1	3.3%	–	–

*Question 5*														
Before	27	90.0%	2	6.7%	1	3.3%	–	–	–	–	–	–	–	–
After	27	90.0%	2	6.7%	1	3.3%	–	–	–	–	–	–	–	–

*Question 6*														
Before	22	73.3%	4	13.3%	4	13.3%	–	–	–	–	–	–	–	–
After	23	76.7%	4	13.3%	3	10.0%	–	–	–	–	–	–	–	–

*Question 7*														
Before	25	83.3%	3	10.0%	1	3.3%	1	3.3%	–	–	–	–	–	–
After	25	83.3%	4	13.3%	–	–	1	3.3%	–	–	–	–	–	–

Question 1, Pressure in the ears; Question 2, Pain in the ears; Question 3, A feeling that your ears are clogged or “under water”; Question 4, Ear problems when you have a cold or sinusitis; Question 5, Crackling or popping sounds in the ears; Question 6, Ringing in the ears; Question 7, A feeling that your hearing is muffled.

**Table 6:** Estimates of the Goodman and Kruskal Gamma (*γ*) coefficients between the answers to the ETDQ-7 questionnaire in the before and after moments.

	Patient + control		Patient		Control	
	Gama (*γ*)	*p*	Gama (*γ*)	*p*	Gama (*γ*)	*p*
Question 1	0.915	<0.001	0.748	<0.001	0.962	<0.001
Question 2	0.915	<0.001	0.915	<0.001	1.000	0.122
Question 3	0.980	<0.001	0.946	<0.001	–	–
Question 4	0.955	<0.001	0.877	<0.001	1.000	<0.001
Question 5	0.951	<0.001	0.835	<0.001	1.000	0.046
Question 6	0.989	<0.001	0.986	<0.001	0.976	<0.001
Question 7	0.954	<0.001	0.846	<0.001	1.000	0.003

Question 1, Pressure in the ears; Question 2, Pain in the ears; Question 3, A feeling that your ears are clogged or “under water”; Question 4, Ear problems when you have a cold or sinusitis; Question 5, Crackling or popping sounds in the ears; Question 6, Ringing in the ears; Question 7, A feeling that your hearing is muffled.

The test–retest reliability assessment was also carried out with the overall score (sum of answers to the seven questions of the ETDQ-7 questionnaire). As it can be observed, the overall score was similar in the “before” and “after” moments (Table 7).

**Table 7:** Summary measures of the overall score obtained in the ETDQ-7 questionnaire, before and after.

	Patient + control (*n* = 50)		Patient (*n* = 20)		Control (*n* = 30)	
	Before	After	Before	After	Before	After
Mean	16.0	15.6	26.9	26.3	8.8	8.4
Median	10.0	9.0	27.0	26.0	8.0	7.0
Minimum	7.0	7.0	10.0	12.0	7.0	7.0
Maximum	48.0	41.0	48.0	41.0	22.0	20.0
Standard-deviation	11.0	10.5	9.5	8.5	3.2	2.8

**Figure 1:**
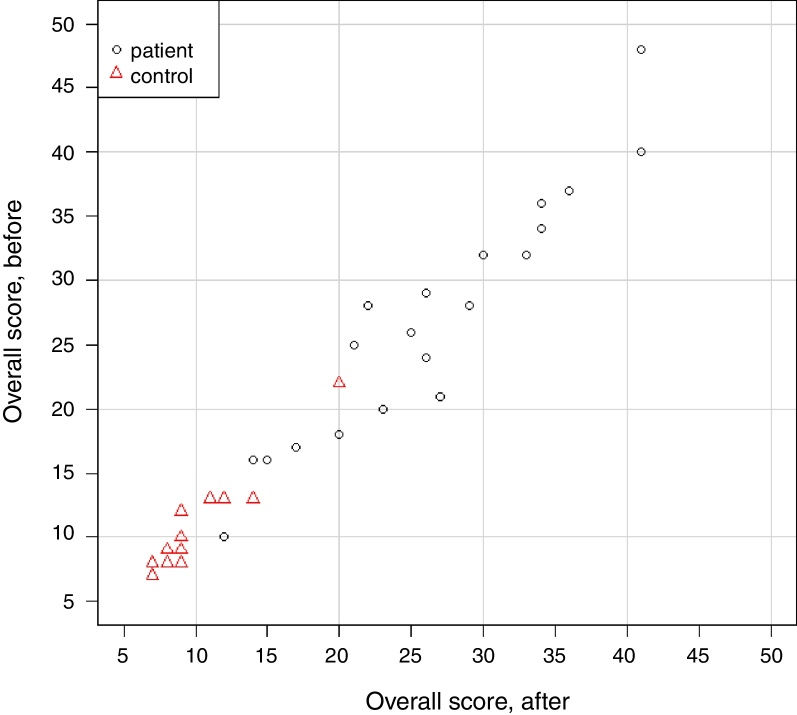
Two-dimensional dispersion diagram of the overall score before and after, according to the group.

Spearman's correlation coefficient (*s*) was also estimated between the overall scores in the “before” and “after” moments. The results showed a strong correlation between the moments, both in the group as a whole (*s* = 0.977, *p* < 0.001) and separately: patient (*s* = 0.943, *p* < 0.001) and control (*s* = 0.913, *p* < 0.001) (Fig. 1).

The questionnaire internal consistency was investigated using Cronbach's alpha, considering all questions and excluding each one of them (Table 8).

**Table 8:** Cronbach's alpha estimates with the exclusion of each question.

	Cronbach's alpha with question exclusion		
	Patient + control (*n* = 50)	Patient (*n* = 20)	Control (*n* = 20)
Question 1	0.676	0.675	0.676
Question 2	0.743	0.698	0.743
Question 3	0.792	0.646	0.792
Question 4	0.639	0.697	0.639
Question 5	0.713	0.766	0.713
Question 6	0.749	0.775	0.749
Question 7	0.750	0.731	0.750

Question 1, Pressure in the ears; Question 2, Pain in the ears; Question 3, A feeling that your ears are clogged or “under water”; Question 4, Ear problems when you have a cold or sinusitis; Question 5, Crackling or popping sounds in the ears; Question 6, Ringing in the ears; Question 7, A feeling that your hearing is muffled.

In brief, considering all the questions, internal consistency was adequate when grouping patients and controls (Cronbach's *α* = 0.762), and also separately: patient (Cronbach's *α* = 0.746) and control (Cronbach's *α* = 0.762).

**Figure 2:**
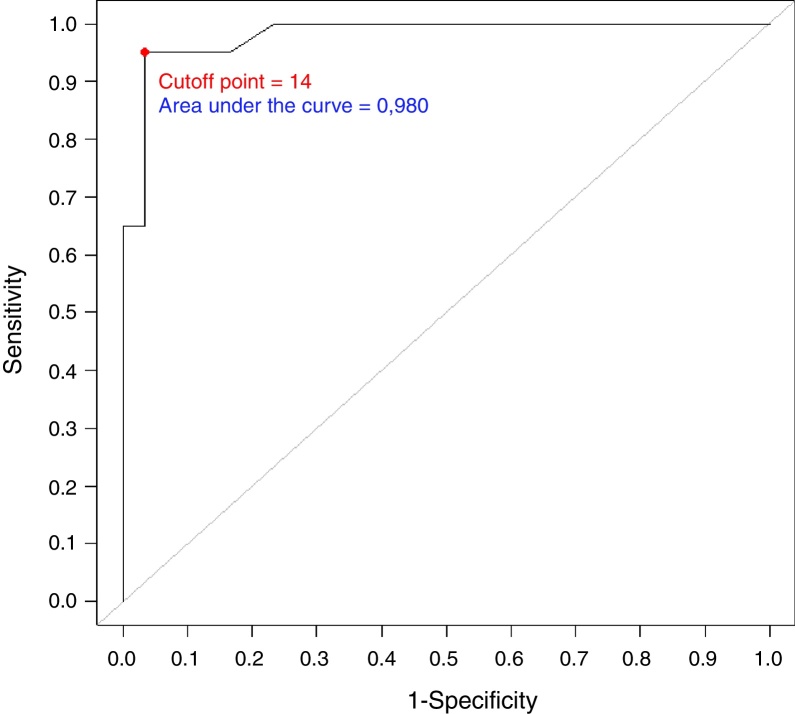
ROC curve considering the overall score at the first moment and group (patient, control).

The questionnaire's discriminatory validity regarding the overall score between patient and control was evaluated through the Receiver Operating Characteristic (ROC) Curve (Fig. 2). The overall score measured at the first moment was used.

Based on information from this curve, we concluded that an overall score ≥14 (cutoff point) has an important discriminatory power between patients and controls (Table 9).

**Table 9:** Distribution of the overall score at the first moment, according to the group.

Overall score	Patient		Control		Patient + control	
≥14	19	95%	1	3.3%	20	66.7%
˂14	1	5%	29	96.7%	30	33.3%
Total	20	100%	30	100%	50	100%

Sensitivity, 19/20 = 0.950; Specificity, 29/30 = 0.967; positive predictive value, 19/20 = 0.950; negative predictive value, 29/30 = 0.967.

## Discussion

Today there is much interest in the Eustachian tube. The concept that the Eustachian tube is not just a static tube, but rather a dynamic organ, has gained acceptance, and with improved clarification of its pathophysiology, new treatments have been developed worldwide. It is agreed that the ET has unique functions and that failure of these functions comprises the syndrome we call “Eustachian Tube Dysfunction” (ETD). ETD is a frequent entity and its chronicity can generate a range of symptoms that affect patient quality of life, which can also lead to infections and hearing loss, leading to an even greater morbidity.^1^, ^4^

In an attempt to improve patient management and follow-up, in 2012 McCoul et al. developed a questionnaire for the evaluation of chronic ETD symptoms consisting of seven items with a scale of responses graded from 1 to 7, with “1” indicating absence of the suggested symptom and “7”, maximum symptom severity. This questionnaire is known as the “Eustachian Tube Dysfunction Questionnaire-7” (ETDQ-7).^8^ The questionnaire was developed with seven items, each dealing with one relevant symptom (most often mentioned in patients’ complaints); it was also designed to be easy to apply and practical. It was modeled after existing questionnaires for the management of patients with chronic otitis and patients with chronic rhinosinusitis, mainly the OM-6 and SNOT 22.

Similar to quality of life questionnaires, symptom scores allow the quantitative measurement of subjective questions and have advantages over the clinical history alone. They provide a formal and valid documentation of patient history as a record and make possible subsequent comparisons after the treatment.^9^

The present study aimed to translate, validate and culturally adapt the ETDQ-7 questionnaire into Brazilian Portuguese. The importance of this study reflects the lack of objective methods to detect and quantify the degree of severity of chronic ETD in adults.^7^ The need for a validated and specific instrument for ETD is particularly remarkable due to the lack of a widely accepted objective measure to detect the presence and severity of this disorder. Several objective measures have been proposed, including audiometry, tympanometry, otoscopy, visual classification of endoscopic findings and tubomanometry. However, no ideal modality has been identified to date.^7^ The availability of a symptom count can help the physician to record an accurate description of the disease condition. The ETDQ-7 is fast and easy to apply. Especially for a country such as Brazil, where the resources are scarce, the questionnaires help us to evaluate the patients at a very low cost.

The criterion validity in our study was established by the presence of normal tympanometry results in control subjects and abnormal ones in individuals with chronic ETD, similar to the original study.

The ETDQ-7 has shown to be reliable and valid for the cross-sectional evaluation of ETD-related symptoms in adults. Particularly, the ability of the ETDQ-7 to discriminate between the patient and non-patient groups was excellent, showing a cutoff point ≥14.5 vs. <14.5 (patients vs. non-patients) with sensitivity and specificity of 100%.

In our study, we demonstrated a cutoff point ≥14 vs. <14 (patients vs. non-patients) with a sensitivity of 95% and specificity of 97%, showing a significant discriminatory power between patients and controls. This minimal difference in our work when compared to the original study may have been caused by the fact that we chose to use an index that gave us an integer as a cutoff, since the scores are given as integers.

In the original study, the retest (questionnaire reapplication) of untreated patients at separate moments (30 days) showed good test–retest reliability, with the retest being performed only in the group with chronic ETD, showing a Spearman's correlation coefficient of 0.78. In our study, Spearman's correlation coefficient was also estimated among the overall scores in the “before” and “after” moments. The results showed a strong correlation between the moments, both in the group as a whole (*s* = 0.977; *p* < 0.001), as well as separately: patient (*s* = 0.943; *p* < 0.001) and control (*s* = 0.913; *p* < 0.001). No medical treatment was prescribed during this time. The patients agreed not to receive treatment during this period, as they were waiting for the surgical procedure (they had previously tried the clinical treatment, without improvement).

The ETDQ-7 internal consistency reliability tests yielded a Cronbach's alpha coefficient of 0.711 in the North-American study for the entire tool (95% Confidence Interval: 0.570–0.818). An internal consistency evaluation after the elimination of each item did not substantially improve the observed internal consistency and, as a result, no items were added or removed from the tool. In our study, the questionnaire's internal consistency was also investigated by Cronbach's alpha estimation, considering all questions and excluding each one, as in the original study.

In short, considering all the questions, internal consistency was adequate for patients and controls together (Cronbach's *α* = 0.762), and also separately: patient (Cronbach's *α* = 0.746) and control (Cronbach's *α* = 0.762). Our results were very similar to those of the original study.

It is noteworthy that our version of ETDQ-7 is not intended to evaluate the symptoms of the Eustachian tube that appear together with acute upper airway infections or neoplastic processes, since these patients were excluded from the study group. All patients had chronic symptoms of ETD and had not received either medical or surgical treatment for their condition, as in the original study.

Some limitations of the ETDQ-7 cited in the original study should be mentioned. Response items are primarily concerned with disease severity. The timing of events, in particular, whether the symptoms are intermittent or continuous, or worsens during a certain time of day, are not represented in most items. Another point to be considered is that the use of tool cannot compare or classify the degrees of severity of the disease among the patients or compare the degree of severity with the tympanometry curve findings, and thus, further studies must be performed to better elucidate these issues. However, it can be of value in comparisons before and after different treatments in the same patient. Finally, the optimal retest period was not determined for the evaluation of ETD symptoms. The retest period for ETDQ-7 was arbitrarily set at one month, although a different recall period might have resulted in different overall responses.

Tools that are specific for disease symptoms can be used as measures for important clinical interventions. Useful attributes that contribute to validity for outcome measurement include responsiveness, sensitivity to clinical change, and criterion validity.

The ETDQ-7 has been translated, validated and adapted to German,^13^ also with similar results to that of the North-American version, and it is strongly suggested as an adjunct method for the diagnosis and management of patients with chronic ETD.

A standardized symptom score can improve clinical management, highlighting the impact of ETD on patient quality of life, helping to guide the adequate treatment, and may also be useful in study comparisons. Further prospective testing of patients undergoing treatment for ETD may establish the usefulness of ETDQ-7 in the evaluation of treatment outcomes.

## Conclusion

The ETDQ-7 was translated, validated and adapted to the Brazilian reality. A standardized symptom score system can improve clinical care, stressing the impact of chronic ETD on the patient's life and guiding adequate patient management.

## Conflicts of interest

The authors declare no conflicts of interest.

## Acknowledgements

To the Coordenação de Aperfeiçoamento de Pessoal de Nível Superior (CAPES, Brazil) for granting a scholarship (Master's Degree) to the main author.
